# Comparison of atazanavir/ritonavir and darunavir/ritonavir based antiretroviral therapy for antiretroviral naïve patients

**DOI:** 10.1186/s12879-017-2379-8

**Published:** 2017-04-11

**Authors:** Tony Antoniou, Leah Szadkowski, Sharon Walmsley, Curtis Cooper, Ann N. Burchell, Ahmed M. Bayoumi, Julio S. G. Montaner, Mona Loutfy, Marina B. Klein, Nima Machouf, Christos Tsoukas, Alexander Wong, Robert S. Hogg, Janet Raboud, The Canadian Observational Cohort (CANOC) collaboration, Robert Hogg, Simon Fraser, Ann N. Burchell, Curtis Cooper, Deborah Kelly, Marina Klein, Mona Loutfy, Nima Machouf, Julio Montaner, Janet Raboud, Chris Tsoukas, Stephen Sanche, Alexander Wong, Tony Antoniou, Ahmed Bayoumi, Mark Hull, Bohdan Nosyk, Angela Cescon, Michelle Cotterchio, Charlie Goldsmith, Silvia Guillemi, P. Richard Harrigan, Marianne Harris, Sean Hosein, Sharon JohnstonClaire Kendall, Clare Liddy, Viviane Lima, David Marsh, David Moore, Alexis Palmer, Sophie Patterson, Peter Phillips, Anita Rachlis, Sean B. Rourke, Hasina Samji, Marek Smieja, Benoit Trottier, Mark Wainberg, Sharon Walmsley, Chris Archibald, Ken Clement, Monique Doolittle-Romas, Laurie Edmiston, Sandra Gardner, Brian Huskins, Jerry Lawless, Douglas Lee, Renee Masching, Stephen Tattle, Alireza Zahirieh, Claire Allen, Stryker Calvez, Guillaume Colley, Jason Chia, Daniel Corsi, Louise Gilbert, Nada Gataric, Katelyn Merritt, Lucia Light, David Mackie, Costa Pexos, Susan Shurgold, Leah Szadkowski, Chrissi Galanakis, Benita Yipv, Jaime Younger, Julia Zhu

**Affiliations:** 1 Keenan Research Centre, Li Ka Shing Knowledge Institute, St. Michael’s Hospital, Toronto, ON Canada; 2 Department of Family and Community Medicine, St. Michael’s Hospital, Toronto, ON Canada; 3 University of Toronto, 410 Sherbourne Street, Toronto, ON ON M4X 1K2 Canada; 4 Toronto General Research Institute, University Health Network, Toronto, ON Canada; 5 The Ottawa Hospital Research Institute, University of Ottawa, Ottawa, ON Canada; 6 British Columbia Centre for Excellence in HIV/AIDS, St. Paul’s Hospital, Vancouver, BC Canada; 7 University of British Columbia, Vancouver, BC Canada; 8 Women’s College Hospital Research Institute, Toronto, ON Canada; 9 Maple Leaf Medical Clinic, Toronto, ON Canada; 10 McGill University Health Centre, McGill University, Montreal, Quebec, Canada; 11 Clinique Médicale l’Actuel, Montreal, Quebec, Canada; 12 Regina Qu’Appelle Health Region, Regina, SK Canada; 13 Simon Fraser University, Burnaby, BC Canada

**Keywords:** HIV, Antiretroviral therapy, Protease inhibitor, Atazanavir, Darunavir

## Abstract

**Background:**

Atazanavir/ritonavir and darunavir/ritonavir are common protease inhibitor-based regimens for treating patients with HIV. Studies comparing these drugs in clinical practice are lacking.

**Methods:**

We conducted a retrospective cohort study of antiretroviral naïve participants in the Canadian Observational Cohort (CANOC) collaboration initiating atazanavir/ritonavir- or darunavir/ritonavir-based treatment. We used separate Fine and Gray competing risk regression models to compare times to regimen failure (composite of virologic failure or discontinuation for any reason). Additional endpoints included virologic failure, discontinuation due to virologic failure, discontinuation for other reasons, and virologic suppression.

**Results:**

We studied 222 patients treated with darunavir/ritonavir and 1791 patients treated with atazanavir/ritonavir. Following multivariable adjustment, there was no difference between darunavir/ritonavir and atazanavir-ritonavir in the risk of regimen failure (adjusted hazard ratio 0.76, 95% CI 0.56 to 1.03) Darunavir/ritonavir-treated patients were at lower risk of virologic failure relative to atazanavir/ritonavir treated patients (aHR 0.50, 95% CI 0.28 to 0.91), findings driven largely by high rates of virologic failure among atazanavir/ritonavir-treated patients in the province of British Columbia. Of 108 discontinuations due to virologic failure, all occurred in patients starting atazanavir/ritonavir. There was no difference between regimens in time to discontinuation for reasons other than virologic failure (aHR 0.93; 95% CI 0.65 to 1.33) or virologic suppression (aHR 0.99, 95% CI 0.82 to 1.21).

**Conclusions:**

The risk of regimen failure was similar between patients treated with darunavir/ritonavir and atazanavir/ritonavir. Although darunavir/ritonavir was associated with a lower risk of virologic failure relative to atazanavir/ritonavir, this difference varied substantially by Canadian province and likely reflects regional variation in prescribing practices and patient characteristics.

## Background

Protease inhibitors remain important options for the treatment of HIV infection [1, 2]. When administered with low doses of ritonavir, protease inhibitors impose a high genetic barrier against the selection of drug-resistant variants of HIV and are therefore especially reliable options for patients for whom poor antiretroviral adherence is anticipated [3, 4]. Because of once-daily dosing and low rates of gastrointestinal adverse effects relative to other members of their class [5, 6], ritonavir-boosted atazanavir and darunavir were, until recently, designated as ‘preferred’ protease inhibitor-based options for the treatment of antiretroviral naïve patients in the United States Depatment of Health and Human Services (DHHS) HIV treatment guidelines [2]. However, atazanavir/ritonavir was reclassified as an ‘alternative’ to darunavir/ritonavir in the most recent iteration of the DHHS guidelines [2] based on high rates of discontinuation due to toxicity among patients treated with atazanavir/ritonavir in ACTG 5257, a randomized trial comparing the efficacy of atazanavir/ritonavir-, darunavir/ritonavir- and raltegravir-based therapy [7].

Despite these findings, questions remain about the comparative effectiveness of atazanavir/ritonavir and darunavir/ritonavir in clinical practice. Although randomized trials are essential for generating evidence about efficacy required to inform clinical guidelines, individuals enrolled in these studies are often not representative of patients treated in routine care [8, 9]. Observational studies can address this limitation by providing evidence of the comparative effectiveness and tolerability of different treatment regimens in patients who are treated in clinical practice. Although observational studies comparing the tolerability of atazanavir/ritonavir and darunavir/ritonavir have been conducted, no such studies have specifically compared the effectiveness of these drugs [10–12]. Accordingly, we conducted a retrospective cohort study comparing the effectiveness and durability of atazanavir/ritonavir- and daruanavir/ritonavir-based regimens among antiretroviral naïve patients enrolled in a longitudinal Canadian cohort study.

## Methods

### Study population

The Canadian Observational Cohort (CANOC) collaboration is a multisite cohort study of antiretroviral-naïve HIV positive patients initiating combination antiretroviral therapy (cART) after January 1, 2000 [13]. The collaboration currently includes 8 participating cohorts from Ontario, Quebec and British Columbia. Criteria for inclusion into CANOC were documented HIV infection, residence in Canada, age 18 years and older, initiation of a first antiretroviral regimen comprised of at least three individual agents, and at least one HIV-1 RNA viral load and CD4 count measurement within one year prior to treatment initiation. Antiretroviral data collection methods vary by site, and include abstraction from patient charts and linkage with provincial prescription databases. Nonnominal data were submitted from each participating site to the coordinating center in Vancouver, British Columbia, Canada.

Participants were eligible for inclusion in this analysis if they initiated atazanavir/ritonavir- or darunavir/ritonavir-based antiretroviral therapy, did not have a viral load less than or equal to 200 copies/mL at or before cART initiation and had at least one follow-up viral load measurement available after treatment initiation. We excluded patients who initiated atazanavir without concomitant ritonavir.

### Outcome measures

The primary outcome of the study was time to regimen failure, defined as a composite of virologic failure and discontinuation for any reason [Table 1, A (includes B1) or B2]. Similar to ACTG 5257, we defined virologic failure as a viral load >1000 copies/mL at or after week 16 but before week 24, or a viral load >200 copies/mL at or after week 24 (A) [7]. Patients who never suppressed or who suppressed and subsequently rebounded were included in this definition. We defined discontinuation as stopping atazanavir or darunavir for more than 60 days. We did not consider changes to the nucleoside backbone, ritonavir, or the addition of other antiretroviral medications as discontinuations. All discontinuations were hierarchically classified as due to virologic failure (B1)or other reasons (B2). Patients who died were considered to have met a competing risk; otherwise patients were censored at the first occurrence of a gap in viral load measurements exceeding two years, the last recorded antiretroviral stop date if followed by a viral load ≤50 copies/mL, or the last available viral load measurement.

**Table 1:** Outcome and Competing Risk Definitions

Outcome	Definition	Competing Risks
Primary Analysis		
Regimen Failure	First A (includes B1) or B2	death
Secondary Analyses		
Virologic Failure	First A (includes B1)	B2, death
Discontinuation Due to Virologic Failure	First B1	B2, death
Discontinuation Due to Other Reasons	First B2	A (includes B1), death
Virologic Suppression	First C	B, death

A. Virologic Failure

B. Discontinuation (hierarchically classified as B1: Due to virologic failure, or B2: Due to other reasons)

C. Virologic Suppression

We conducted several secondary analyses (Table 1). First, we examined time to virologic failure (A) and time to discontinuations due to virologic failure (B1) separately, considering death and discontinuations for other reasons (B2) as competing risks. Next, in the absence of specific adverse event data, we examined time to discontinuation of either darunavir or atazanavir for reasons other than virologic failure (B2). Such discontinuations may have occurred because of toxicities attributable to the protease inhibitor. Individuals who experienced virologic failure (A) and those who died were considered to have met a competing risk. Finally, we examined time to virologic suppression (C), defined as time to the first of at least two consecutive viral load measurements below 50 copies/mL at least 30 days apart. Patients who died or discontinued atazanavir or darunavir for any reason (B) were considered to have met a competing risk. We used the same censoring rules as in the primary analysis in all secondary analyses.

### Statistical analysis

All analyses were conducted with SAS 9.4 (SAS Institute, Cary, North Carolina, USA) and R 3.3.1 (R Development Core Team, Vienna, Austria). We compared baseline characteristics between the two regimen groups using chi-square tests for categorical variables and Wilcoxon rank sum tests for continuous variables. For each outcome, cumulative incidence functions taking into account competing risks were determined by regimen and compared using Gray’s Test for Equality [14].

We used multivariable Fine and Gray competing risk regression [15] to estimate the association between treatment with darunavir/ritonavir relative to atazanavir/ritonavir and each outcome. We adjusted our models for age, sex and men who have sex with men (MSM) status, race, baseline viral load and CD4 count, calendar year of treatment initiation, nucleoside analogue backbone (emtricitabine/tenofovir versus other backbones), Canadian province of residence, and history of injection drug use (IDU). Because of collinearity beween IDU and co-infection with hepatitis C, we adjusted our models for the former variable only. For covariates with large amounts of missing data, separate categories for missing were created when these variables were included in the regression models.

We conducted several sensitivity analyses to test the robustness of our findings. First, we conducted analyses by provincial subgroup (British Columbia vs Ontario/Quebec) because of regional differences in prescribing patterns and characteristics of CANOC participants [13]. Specifically, antiretroviral treatment guidelines in British Columbia recommended efavirenz or atazanavir/ritonavir as first-line regimens during our study period, favouring the latter for patients who use drugs or with mental health illness and preserving darunavir/ritonavir for treatment failures. In contrast, no such recommendations were in place in Ontario and Quebec during the study period. Further, participants in British Columbia differ from those in Ontario and Quebec in important ways, including mode of HIV acquisition and hepatitis C coinfection [13]. We therefore reasoned that residual confounding due to associated unmeasured variables such as mental health illness and socioeconomic status could occur. Second, we examined whether outcomes varied by baseline viral load (< 100,000 copies/mL or >100,000 copies/mL). Third, because approximately one-quarter of atazanavir-treated patients started treatment between 2004 and 2006, we replicated our analyses, restricting to patients who started treatment on or after January 1, 2010. Fourth, patients who had a viral load of ≤50 copies/mL and who were switched to a single table regimen were re-defined as having discontinued treatment for simplification and included as competing risks. Finally, we replicated our analyses following multiple imputation for missing values of the race, IDU and MSM variables. To our knowledge, no methods currently exist to impute data specifically for Fine & Gray competing risk regression models, and so a substantive model compatible version of fully conditional specification was used to impute data intended for cause-specific competing risk regression models [16]. For each outcome, all covariates used in the original multivariable models were used to impute missing values five different times. Fine & Gray models were then run on each imputed dataset and the resulting parameter estimates and variances were combined using Rubin’s rules [17, 18].

## Results

During the study period, we identified 1791 eligible patients whose first cART regimen included atazanavir/ritonavir and 222 eligible patients whose first regimen included darunavir/ritonavir. Patients treated with atazanavir/ritonavir were more likely to be female (19% vs. 13%; *p* = 0.02), co-infected with hepatitis C (30% vs. 13%; *p* < 0.0001) and report injection drug use as a risk factor for HIV infection (29% versus 9%; *p* < 0.0001) (Table 2). These differences were mitigated when restricting comparisons to participants from Ontario and Quebec only. The median (interquartile range, IQR) duration of follow-up was 3.5 (1.6, 5.4) years and 1.5 (0.7, 2.3) years in the atazanavir/ritonavir and darunavir/ritonavir treated participants respectively (Table 2).

**Table 2:** Demographic and clinical characteristics at initiation of combination antiretroviral therapy by regimen^a^

	Overall Sample				Ontario & Quebec Sub-analysis		
	Total *N* = 2013	Atazanavir *N* = 1791	Darunavir *N* = 222	*p*	Atazanavir *N* = 544	Darunavir *N* = 183	*p*
Demographics							
Age	40 (33–47)	40 (33–47)	40 (32–46)	0.25	38 (32–45)	39 (32–46)	0.49
< 35	601 (30%)	521 (29%)	80 (36%)	0.03	189 (35%)	69 (38%)	0.02
35–44	746 (37%)	680 (38%)	66 (30%)		217 (40%)	53 (29%)	
≥ 45	666 (33%)	590 (33%)	76 (34%)		138 (25%)	61 (33%)	
Male	1632 (81%)	1439 (81%)	193 (87%)	0.02	434 (80%)	162 (89%)	<.01
Gender & MSM							
Female	373 (19%)	344 (19%)	29 (13%)	<.0001	110 (20%)	21 (11%)	<.01
MSM	764 (38%)	646 (36%)	118 (53%)		293 (54%)	113 (62%)	
Male, not MSM	479 (24%)	451 (25%)	28 (13%)		93 (17%)	22 (12%)	
Missing	397 (20%)	350 (20%)	47 (21%)		48 (9%)	27 (15%)	
Race							
White	531 (26%)	492 (27%)	39 (18%)	<.001	146 (27%)	34 (19%)	<.0001
Black	128 (6%)	117 (7%)	11 (5%)		84 (15%)	11 (6%)	
Aboriginal Peoples	109 (5%)	101 (6%)	8 (4%)		11 (2%)	7 (4%)	
Other	145 (7%)	133 (7%)	12 (5%)		41 (8%)	9 (5%)	
Missing	1100 (55%)	948 (53%)	152 (68%)		262 (48%)	122 (67%)	
Injection drug use							
No	1207 (60%)	1053 (59%)	154 (69%)	<.0001	426 (78%)	142 (78%)	0.03
Yes	542 (27%)	521 (29%)	21 (9%)		59 (11%)	11 (6%)	
Missing	264 (13%)	217 (12%)	47 (21%)		59 (11%)	30 (16%)	
Endemic country^b^							
No	537 (27%)	406 (23%)	131 (59%)	<.0001	406 (75%)	131 (72%)	0.02
Yes	99 (5%)	80 (4%)	19 (9%)		80 (15%)	19 (10%)	
Missing	1377 (68%)	1305 (73%)	72 (32%)		58 (11%)	33 (18%)	
Province							
British Columbia	1286 (64%)	1247 (70%)	39 (18%)	<.0001	--	--	
Ontario	355 (18%)	264 (15%)	91 (41%)		264 (49%)	91 (50%)	0.78
Quebec	372 (18%)	280 (16%)	92 (41%)		280 (51%)	92 (50%)	
Clinical							
Hepatitis C							
No	1349 (67%)	1168 (65%)	181 (82%)	<.0001	449 (83%)	155 (85%)	0.75
Yes	559 (28%)	530 (30%)	29 (13%)		71 (13%)	20 (11%)	
Missing	105 (5%)	93 (5%)	12 (5%)		24 (4%)	8 (4%)	
CD4 count (cells/mm^3^)	220 (120–330)	220 (120–320)	280 (130–370)	<.01	230 (149–310)	286 (130–370)	<.01
<200	869 (43%)	795 (44%)	74 (33%)	<.001	214 (39%)	60 (33%)	<.0001
200–349	693 (34%)	620 (35%)	73 (33%)		234 (43%)	60 (33%)	
350–499	287 (14%)	237 (13%)	50 (23%)		69 (13%)	45 (25%)	
≥500	164 (8%)	139 (8%)	25 (11%)		27 (5%)	18 (10%)	
Log 10 (VL copies/mL)	4.91 (4.41–5.11)	4.90 (4.42–5.05)	4.94 (4.36–5.44)	0.03	4.82 (4.35–5.26)	4.90 (4.26–5.39)	0.43
VL ≥ 100,000 copies/mL	881 (44%)	780 (44%)	101 (45%)	0.58	209 (38%)	77 (42%)	0.38
Year of cART initiation	2009 (2007–2010)	2008 (2006–2010)	2011 (2010–2011)	<.0001	2008 (2007–2009)	2010 (2010–2011)	<.0001
2003–2006	474 (24%)	473 (26%)	1 (0%)	<.0001	121 (22%)	1 (1%)	<.0001
2007–2009	799 (40%)	767 (43%)	32 (14%)		307 (56%)	32 (17%)	
2010–2012	740 (37%)	551 (31%)	189 (85%)		116 (21%)	150 (82%)	
First NRTIs							
3TC/ABA	499 (25%)	441 (25%)	58 (26%)	<.0001	259 (48%)	53 (29%)	<.0001
3TC/TDF	285 (14%)	284 (16%)	1 (0%)		36 (7%)	1 (1%)	
FTC/TDF	1172 (58%)	1009 (56%)	163 (73%)		230 (42%)	129 (70%)	
Other	57 (3%)	57 (3%)	0 (0%)		19 (3%)	0 (0%)	
Follow up							
Years of Follow up	3.10 (1.45–5.16)	3.50 (1.63–5.38)	1.48 (0.69–2.25)	<.0001	3.73 (2.20–5.32)	1.68 (0.86–2.46)	<.0001
VLs per year	4.67 (3.61–6.21)	4.68 (3.63–6.16)	4.50 (3.55–6.56)	0.71	3.88 (3.12–4.76)	4.32 (3.44–5.76)	<.001
0–3	274 (14%)	246 (14%)	28 (13%)	0.43	121 (22%)	26 (14%)	<.001
3–6	1187 (59%)	1062 (59%)	125 (56%)		358 (66%)	115 (63%)	
> 6	552 (27%)	483 (27%)	69 (31%)		65 (12%)	42 (23%)	

*MSM* men who have sex with men, *VL* viral load, *cART* combination antiretroviral therapy, *NRTI* nucleoside reverse transcriptase, *3TC* lamivudine, *ABC* abacavir, *TDF* tenofovir, *FTC* emtricitabine

a Medians (IQRs) are presented for continuous variables and frequencies (percent) for categorical variables

b Indicates patients who have immigrated to Canada from a country with a high prevalence of HIV

**Fig. 1:**
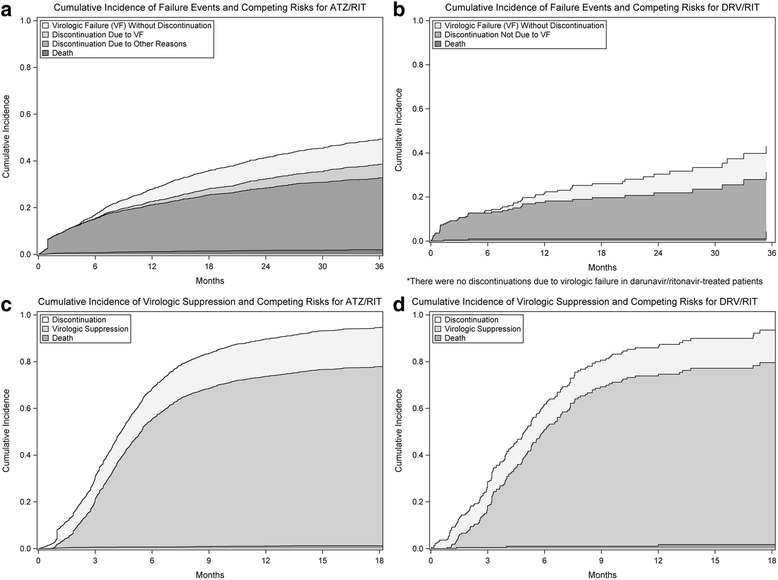
Cumulative incidence functions of time to events and competing risks

The cumulative incidence of regimen failure (A or B2) one year following the initiation of treatment was 0.29 (95% confidence interval (CI) 0.27 to 0.31) for patients initiating atazanavir/ritonavir and 0.21 (95% CI 0.15 to 0.27) for those starting darunavir/ritonavir (*p* < 0.01, Fig. 1a and b). A total of 43 patients experienced a competing risk of death. Following multivariable adjustment, there was no difference between darunavir/ritonavir and atazanavir-ritonavir in the risk of regimen failure (adjusted hazard ratio 0.76, 95% CI 0.56 to 1.03) (Table 3). This finding was similar in sensitivity analyses (Table 4).

**Table 3:** Multivariable Fine and Gray models of time to events

	Virologic Failure or Other Discontinuation		Virologic Failure		Discontinuation due to Virologic Failure		Other Discontinuation		Virologic Suppression	
	aHR (95% CI)	*p*	aHR (95% CI)	*p*	aHR (95% CI)	*p*	aHR (95% CI)	*p*	aHR (95% CI)	*p*
DRV/RIT	0.76 (0.56,1.03)	0.08	0.50 (0.28,0.91)	0.02	0.00 (0.00,0.00)	<.0001	0.93 (0.65,1.33)	0.68	0.99 (0.82,1.21)	0.96
FTC/TEN	1.02 (0.86,1.21)	0.84	1.30 (0.99,1.70)	0.06	1.41 (0.87,2.28)	0.16	0.87 (0.70,1.08)	0.20	0.99 (0.87,1.12)	0.82
Age (per 10y)	0.89 (0.83,0.95)	<.001	1.02 (0.91,1.15)	0.70	0.89 (0.73,1.09)	0.27	0.86 (0.79,0.94)	<.001	1.04 (0.98,1.10)	0.17
Gender & MSM										
Female	Ref.		Ref.		Ref.		Ref.		Ref.	
MSM	0.68 (0.55,0.84)	<.001	0.73 (0.51,1.03)	0.08	0.46 (0.24,0.89)	0.02	0.79 (0.61,1.03)	0.08	1.20 (1.00,1.45)	0.05
Male, not MSM	0.72 (0.59,0.88)	<.01	0.90 (0.64,1.25)	0.51	0.70 (0.42,1.17)	0.17	0.76 (0.59,0.98)	0.03	1.06 (0.87,1.28)	0.57
Missing	0.74 (0.56,0.98)	0.03	0.80 (0.47,1.35)	0.40	0.64 (0.27,1.55)	0.33	0.83 (0.60,1.15)	0.26	1.13 (0.90,1.42)	0.31
Race										
White	Ref.		Ref.		Ref.		Ref.		Ref.	
Black	0.89 (0.64,1.23)	0.47	1.41 (0.88,2.26)	0.15	1.18 (0.47,2.95)	0.72	0.64 (0.40,1.04)	0.07	1.16 (0.88,1.54)	0.29
Aboriginal Peoples	1.39 (1.05,1.85)	0.02	1.14 (0.72,1.82)	0.57	1.46 (0.71,2.99)	0.30	1.32 (0.91,1.89)	0.14	0.68 (0.51,0.92)	0.01
Other	0.89 (0.67,1.18)	0.42	1.04 (0.68,1.59)	0.86	0.79 (0.36,1.75)	0.56	0.82 (0.56,1.19)	0.29	1.15 (0.94,1.40)	0.17
Missing	1.17 (0.98,1.39)	0.08	0.75 (0.55,1.01)	0.06	0.70 (0.43,1.14)	0.15	1.38 (1.12,1.72)	<.01	0.93 (0.80,1.08)	0.35
IDU										
No	Ref.		Ref.		Ref.		Ref.		Ref.	
Yes	1.68 (1.39,2.02)	<.0001	1.46 (1.05,2.04)	0.03	1.64 (0.92,2.94)	0.09	1.50 (1.20,1.88)	<.001	0.69 (0.58,0.82)	<.0001
Missing	1.11 (0.83,1.49)	0.48	1.16 (0.68,1.96)	0.58	1.10 (0.41,2.93)	0.85	1.04 (0.74,1.45)	0.83	0.91 (0.72,1.14)	0.40
Province										
BC	Ref.		Ref.		Ref.		Ref.		Ref.	
ON	0.95 (0.77,1.16)	0.60	1.11 (0.81,1.54)	0.52	0.67 (0.34,1.31)	0.24	0.90 (0.69,1.17)	0.44	0.97 (0.82,1.15)	0.73
QC	0.82 (0.66,1.02)	0.08	1.26 (0.88,1.80)	0.21	1.03 (0.51,2.08)	0.93	0.68 (0.51,0.90)	<.01	1.02 (0.86,1.21)	0.83
Baseline CD4 (per 100)										
< 200	Ref.		Ref.		Ref.		Ref.		Ref.	
200–349	0.83 (0.71,0.96)	0.01	0.93 (0.73,1.20)	0.58	1.16 (0.76,1.77)	0.48	0.84 (0.69,1.02)	0.08	1.18 (1.04,1.33)	<.01
350–499	0.80 (0.63,1.01)	0.06	0.52 (0.33,0.84)	<.01	0.60 (0.23,1.56)	0.29	1.00 (0.76,1.31)	0.99	1.35 (1.14,1.61)	<.001
≥ 500	1.07 (0.80,1.45)	0.64	0.79 (0.44,1.42)	0.44	0.95 (0.33,2.69)	0.92	1.22 (0.87,1.71)	0.25	1.36 (1.08,1.71)	<.01
Baseline VL ≥ 100,000	1.20 (1.05,1.38)	<.01	1.74 (1.38,2.20)	<.0001	2.27 (1.49,3.48)	<.001	0.95 (0.80,1.13)	0.57	0.61 (0.55,0.68)	<.0001
Calendar Year										
2003–2006	Ref.		Ref.		Ref.		Ref.		Ref.	
2007–2009	0.96 (0.79,1.17)	0.69	0.68 (0.50,0.92)	0.01	0.41 (0.24,0.71)	<.01	1.11 (0.87,1.42)	0.39	0.96 (0.82,1.12)	0.58
2010–2012	1.12 (0.88,1.42)	0.35	0.88 (0.61,1.28)	0.50	0.64 (0.33,1.24)	0.19	1.03 (0.77,1.38)	0.84	0.67 (0.56,0.81)	<.0001

*AHR* adjusted hazard ratio, *DRV/r* darunavir/ritonavir, *ATZ/r* atazanavir/ritonavir, *FTC* emtricitabine, *TDF* tenofovir, *NRTI* nucleoside reverse transcriptase inhibitor, *MSM* men who have sex with men, *VL* viral load

**Table 4:** Adjusted hazard ratios for darunavir/ritonavir vs. atazanavir/ritonavir from sensitivity analyses

Analysis		Virologic Failure or Other Discontinuation		Virologic Failure		Discontinuations due to Virologic Failure		Other Discontinuation		Virologic Suppression	
		aHR (95% CI)	*p*	aHR (95% CI)	*p*	aHR (95% CI)	*p*	aHR (95% CI)	p	aHR (95% CI)	*p*
0	Original Model	0.76 (0.56,1.03)	0.08	0.50 (0.28,0.91)	0.02	0.00 (0.00,0.00)	<.0001	0.93 (0.65,1.33)	0.68	0.99 (0.82,1.21)	0.96
1	Subgroup by Province										
	ON/QC Only	0.73 (0.51,1.06)	0.10	0.90 (0.44,1.86)	0.78	0.00 (0.00,0.00)	<.0001	0.72 (0.47,1.11)	0.14	0.99 (0.78,1.27)	0.96
	BC Only	0.69 (0.34,1.42)	0.32	0.41 (0.09,1.78)	0.23	0.00 (0.00,0.00)	<.0001	0.93 (0.43,2.04)	0.86	0.78 (0.46,1.32)	0.35
2	Subgroup by Baseline VL										
	< 100,000 copies/mL	0.66 (0.42,1.03)	0.07	0.47 (0.18,1.19)	0.11	0.00 (0.00,0.00)	<.0001	0.82 (0.49,1.36)	0.44	0.97 (0.76,1.23)	0.78
	≥ 100,000 copies/mL	0.86 (0.56,1.33)	0.49	0.54 (0.25,1.16)	0.11	0.00 (0.00,0.00)	<.0001	1.16 (0.68,2.00)	0.58	1.11 (0.81,1.53)	0.51
3	2010–2012 Only	0.81 (0.56,1.18)	0.27	0.55 (0.26,1.14)	0.11	0.00 (0.00,0.00)	<.0001	0.96 (0.63,1.48)	0.87	0.98 (0.76,1.26)	0.87
4	Simplification discontinuations included as competing risks	0.75 (0.54,1.03)	0.07	0.50 (0.28,0.91)	0.02	0.00 (0.00,0.00)	<.0001	0.92 (0.63,1.34)	0.65	0.99 (0.82,1.21)	0.96
5	SMC-FCS Imputation	0.76 (0.56, 1.04)	0.08	0.5 (0.28, 0.9)	0.02	0.00 (0.00,0.00)	<.0001	0.91 (0.62, 1.34)	0.64	0.98 (0.8, 1.2)	0.84

*aHR* adjusted hazard ratio, *VL* viral load

The one-year cumulative incidence of virologic failure (A) was 0.09 (95% CI 0.08 to 0.10) for patients starting atazanavir/ritonavir and 0.04 (95% CI 0.02 to 0.08) for those starting darunavir/ritonavir (*p* = 0.02, Fig. 1a and b). Forty-three patients died prior to virologic failure. Following multivariable adjustment, the risk of virologic failure was lower in darunavir/ritonavir-treated patients (adjusted hazard ratio 0.50, 95% CI 0.28 to 0.91) (Table 3). However, this finding was driven primarily by high failure rates among atazanavir/ritonavir-treated patients in the province of British Columbia. In a sensitivity analysis by Canadian province, there was no difference in the risk of virologic failure among participants in Ontario and Quebec (adjusted hazard ratio 0.90, 95% CI 0.44 to 1.86) (Table 4). In the analysis of participants from British Columbia, the point estimate of the adjusted hazard ratio of virologic failure associated with darunavir was similar (adjusted hazard ratio = 0.41, 95% CI (0.09, 1.78)) to that from the analysis of the entire cohort but the finding was not statistically significant due to the infrequent use of darunavir in this province (Table 4).

The cumulative incidence of discontinuation due to virologic failure (B1) in atazanavir/ritonavir and darunavir/ritonavir-treated patients was 0.01 (95% CI 0.01 to 0.02) and 0.00, respectively (*p* = 0.01, Fig. 1a and b). The lack of events in darunavir/ritonavir treated patients resulted in adjusted hazard ratios of 0.00 for darunavir/ritonavir vs. atazanavir/ritonavir when modelling this outcome.

The cumulative incidence of discontinuation for reasons other than virologic failure (B2) was 0.2 (95% CI 0.18 to 0.22) for patients initiating atazanavir/ritonavir and 0.17 (95% CI 0.12 to 0.22) for patients initiating darunavir/ritonavir (*p* = 0.16, Fig. 1a and b). Forty-three patients experienced a competing risk of death. There was no difference between darunavir/ritonavir and atazanavir/ritonavir in the risk of discontinuation for reasons other than virologic failure after adjusting for confounding variables (adjusted hazard ratio 0.93, 95% CI 0.65 to 1.33) (Table 3). These results were similar in sensitivity analyses (Table 4).

The cumulative incidence of virologic suppression (C) at 1 year after treatment initiation was 0.73 (95% CI 0.70 to 0.75) for participants whose first regimen included atazanavir/ritonavir and 0.73 (95% CI 0.66 to 0.79) for those whose first regimen included darunavir/ritonavir (*p* = 0.62, Fig. 1c and d). Twenty-four patients died prior to achieving virologic suppression. Following multivariable adjustment, there was no difference in the time to virologic suppression (adjusted hazard ratio 0.99, 95% CI 0.82 to 1.21) according to treatment regimen (Table 3). Results were similar in sensitivity analyses (Table 4).

In sensitivity analyses of patients who initiated treatment on or after January 1, 2010, the adjusted hazards ratios for treatment group were very similar to those of the main analysis considering patients starting on or after January 1, 2003 (Table 4). However, the estimates were less precise because of the smaller sample size.

## Discussion

In our analysis of more than two thousand antiretroviral naïve patients from three Canadian provinces, patients initiating darunavir/ritonavir were at a lower risk of virologic failure (A) and subsequent discontinuations due to virologic failure (B1) than patients treated with atazanavir/ritonavir. In contrast, we observed no differences in time to regimen failure (A or B2), virologic suppression (C) or to discontinuation for reasons other than virologic failure (B2).

Our findings differ from those of ACTG 5257, a randomized trial comparing darunavir/ritonavir, atazanavir/ritonavir- and raltegravir-based antiretroviral therapy [7]. Specifically, this trial found a higher incidence of tolerability discontinuation among patients randomized to atazanavir/ritonavir, mediated primarily by participant-driven regimen change for jaundice or hyperbilirubinemia and non-hepatobiliary gastrointestinal side effects. Although we did not have data regarding the exact reasons for discontinuation, we found no difference in the risk of discontinuations for reasons other than virologic failure (B2), an outcome which includes toxicity-driven discontinuation. In a sensitivity analysis, there was no difference in the risk of discontinuations for reasons other than (i) virologic failure or (ii) simplification, an outcome that may more closely approximate toxicity-driven discontinuation. The discrepancy between the results of our analysis and those of the ACTG 5257 trial may be due in part to differences in the calendar year periods of atazanavir and darunavir initiation in our study. Because atazanavir-treated patients started treatment approximately three years earlier than darunavir-treated patients, with approximately one-quarter starting between 2004 and 2006, we speculated that discontinuations due to jaundice or asymptomatic hyperbilirubinemia were tempered by a lack of potent, tolerable treatment alternatives during this period. Most notably, 70% of patients starting atazanavir/ritonavir resided in British Columbia, a province where the epidemic is driven primarily by injection drug use, thereby potentially deterring clinicians from switching patients to regimens with lower barriers to resistance or higher rates of gastrointestinal side effects. In contrast, ACTG 5257 participants initiated treatment between 2009 and 2011, a period marked by the availability of potent treatment alternatives conducive to supporting participant-driven requests for regimen change. Although sensitivity analyses restricted to participants initiating cART between 2010 and 2012 yielded similar results, we speculate that this reflects a desire to preserve darunavir/ritonavir and other new options as second-line regimens for patients treated with atazanavir/ritonavir in British Columbia.

Our finding of an increased risk of virologic failure among patients receiving atazanavir/ritonavir also contrasts with the results of ACTG 5257, in which no difference was observed in this outcome. A possible explanation relates to inter-study differences in the prevalence and distribution of baseline characteristics known to adversely affect adherence and sustained virologic suppression. Specifically, relative to ACTG 5257 our study population included a greater proportion of patients who had a history of injection drug use (27% versus 7%), were coinfected with hepatitis C (28% versus 7.8%) and with baseline CD4 counts below 350 cells/mm^3^ (77% versus 8.8%). Notably, these characteristics were disproportionately represented among patients treated with atazanavir/ritonavir in our study, likely predisposing these patients to virologic failure.

Inter-provincial differences in patient characteristics and prescribing practices may also account for disparate findings between our study and ACTG 5257. Specifically, a higher prevalence of injection drug use and hepatitis C was observed among participants in British Columbia relative to Ontario and Quebec. Although we adjusted for injection drug use in our analysis, we lacked data regarding co-existing mental health illness, other substance use and socioeconomic status; residual confounding is therefore possible. Also, as noted earlier, atazanavir/ritonavir was the recommended protease inhibitor for antiretroviral naïve patients in British Columbia during the study period, particularly for patients with co-morbid illness or social circumstances that could predispose them to treatment failure. In contrast, no such recommendations were in place in Ontario and Quebec during the study period. The impact of these selection biases was evident in sensitivity analyses restricted to participants from Ontario and Quebec, in that no differences between regimens were observed for any outcome, including virologic failure.

Strengths of our analysis include the size of the study population, the diversity of the participants in CANOC and the ability to compare clinical outcomes between individuals initiating atazanavir/ritonavir and darunavir/ritonavir in a clinical practice setting. However, several limitations of our study merit emphasis. Most notably, as with all observational studies, our findings may be biased by residual inter-group differences in baseline variables and unmeasured confounders. In addition, as noted earlier, differences in the timing of darunavir and atazanavir availability may have precluded our ability to observe differences in the risk of treatment discontinuation. The nature of antiretroviral data in a retrospective cohort also imposes some limitations when considering discontinuation. Some sites have prescription based data with stop dates calculated from the days supplied by the prescription. These stop dates are inaccurate when a patient is not perfectly adherent. Other sites provide start and stop dates abstracted from patient charts which are subject to recall and documentation errors. Since 70% of participants who had atazanavir as part of their first regimen resided in British Columbia, where antiretroviral records are prescription-based compared to 18% of participants who had darunavir as part of their starting regimen, this may have resulted in an overestimate of time to discontinuation among participants on atazanavir. Finally, we lacked data regarding exact reasons for treatment discontinuation.

## Conclusions

In conclusion, darunavir/ritonavir and atazanavir/ritonavir were of similar effectiveness in the treatment of antiretroviral naïve patients. Differences in the risk of virologic failure between darunavir/ritonavir and atazanavir/ritonavir varied substantially by province due to regional differences in prescribing patterns and patient characteristics. Our data provide both a comparison of the effectiveness of these two protease inhibitors in the clinical setting and an illustration of the potential magnitude and impact of selection bias in a cohort study setting.

## Abbreviations

CANOC: Canadian observational cohort
CART: Combination antiretroviral therapy
CI: Confidence interval
DHHS: Department of health and human services
IDU: Injection drug use
IQR: Interquartile range
MSM: Men who have sex with men
